# Physical restraining: Nurses knowledge and practice in Tertiary Care Hospital of Eastern Nepal

**DOI:** 10.1002/nop2.298

**Published:** 2019-06-12

**Authors:** Nirmala Pradhan, Sami Lama, Gayananda Mandal, Erina Shrestha

**Affiliations:** ^1^ B.P. Koirala Institute of Health Sciences Dharan Nepal

**Keywords:** nurses roles, nursing intervention, physical restraining

## Abstract

**Aim:**

The aim was to assess the level of knowledge and practice on physical restraints.

**Design:**

A descriptive cross‐sectional study design was adopted.

**Methods:**

A total enumerative sampling technique was used to select 117 nurses working in ICU, medical wards, psychiatric ward and emergency unit of Tertiary Care Hospital, Nepal. After obtaining written consent from each participant, self‐administered questionnaires on socio‐demographic profile, knowledge and practice about physical restraints were distributed.

**Results:**

Most nurses (55.6%) were from age group of 18–25 years. Most of the participants (82.1%) had completed their Diploma Nursing, and 33.3% of the nurses were working in the ICU. Maximum of the participants (74.4%) had previous exposure to physical restraints. In knowledge of physical restraints, the score of 52.1% showed above the median range (Median [IQR] = 43 [54–30]). In the case of practice, 54.7% of the nurses showed adequate practice (mean = 80.1, *SD* 7.7).

## 1 INTRODUCTION

Physical restraint refers to any physical method of restricting a person's freedom of movement, physical activity or normal access to his or her body. In hospitals, physical restraints were used primarily to prevent falls and stop confused patients from wandering and harming themselves (Sonya & Negm, 2013). Preventing and protecting the patient from harm are central nursing responsibilities for individuals who are temporarily incapacitated.

Nurses are the key decision‐makers in the application of physical restraints to patients. However, a significant number of nurses continue to hold misconceptions about their proper use (Huang, 2003). Physical restraint includes the following measures “Postural” restraint as a way to help to maintain a correct position (e.g., as part of a postural re‐education treatment); “Active” restraint, set, most often, by a physical therapist (e.g., to prepare the vertical position after a long period in bed); “Passive” restraint, which constitutes the use of all the means, methods, materials or garments that prevent or limit voluntary mobility capacity of the whole body or a portion thereof, having the purpose of the patient's safety (Cunha, Andre, Bica, Ribeiro, & Dias, 2016).

Aggressive or disruptive behaviour of a patient may appear in an unexpected way and, as soon as it arises, whenever it is possible, a verbal approach as a priority form of containment should be attempted. The resort to physical restraint should only be used in case this approach is not effective. But caution should be taken which avoid physical or psychological damages of the people and the people nearby.

Aggressive situations may be associated with behavioural changes, like psychomotor agitation, dementia, previous trauma, psychotic disorders including delusions, hallucinations (e.g., schizophrenia), mood and personality disorders. In this context, since nurses are a group of professionals whose functions require greater proximity to the patient. It is a challenge to develop assertive nursing practices that are efficient enough to ensure the customer integrity (Cunha et al., 2016).

Moreover, violent and aggressive behaviour in patients can be triggered by environmental and contextual factors. Unclear policy and guidelines, overcrowding, poor ward design, inexperienced staff, poor staff retention and poor information sharing contribute to violent or aggressive behaviour. Studies have also shown a link between staff characteristics and the development of aggression and violence in mental health patients (Duxbury & Wright, 2011).

## 2 BACKGROUND

Patients’ aggression in healthcare settings continues to be a matter of concern, raising questions over the safety of both patients and staff. In 2005, in the UK the audit found that violence against nursing staff was “consistently high” with up to 86% of nurses being affected (Duxbury & Wright, 2011).

Previous studies have evidenced adverse effects of physical restraint, that is skin trauma, pressure sores, muscular atrophy, nosocomial infection, constipation, incontinence, limb injury, contractures, depression, anger and a decline in functional and cognitive state and increasing agitation (Evans, 2002). Also, it was reported that use of physical restraint resulted in negative effects on patients and their families, particularly on the patients feeling disgraced and embarrassed in remembering the experience (Duxbury & Wright, 2011). This is due to negative feelings caused by feeling of confinement, loss of dignity and identity, aggression, social isolation and anxiety. On the other hand, nursing staff may have feelings of guilt and frustration when they must restrain a patient (Gastmans & Milisen, 2006).

A recent cross‐sectional study conducted by Eskandari, Abdullah, Zainal, and Wong (2017), on use of physical restraint, showed moderate knowledge and attitude with strong intention to use physical restraint was found among the nurses. Less than half of the nurses considered alternatives to physical restraint and most of them did not understand the reasons for the physical restraint. Similarly, a study conducted in a Turkish hospital on physical restraint reported nine deaths of patients in chest restraints (Demir, 2007). Furthermore, it was reported in a similar study done in Hong Kong that nurses had inadequate knowledge and negative attitudes towards the staff (Suen, Lai, & Wong, 2006). Despite extensive literature on the potential complications of using physical restraint, it is still considered as a permanent and effective intervention in the management of unpleasant behaviours on acute and long‐term care environments.

Managing aggressive and violent behaviours has become an essential skill and important to all involved with psychiatric patients. Much evidence that has been collected demonstrates that behavioural approaches to care can provide effective alternatives to reliance on restraint (Duxbury & Wright, 2011). Successful strategies such as clear guidelines and a comprehensive reporting requirement commitment by management, adequate staffing levels and staff training in the safe use of, alternatives to, restraints are keys to prevention. Proper training increases the behavioural competence of all direct care staff, while administrative increases the behavioural competence encourages the competent application of behavioural skills and ensures effective oversight by those who are relatively more competent (Judy, Regan, Kerri, Uzma, & Wright, 2006).

To determine the meaning of the patient's behaviour is the first step to achieve the goal and, possibly, the most difficult for health professionals. And although the physical restraints are used to “protect” or “help” sick people, they are seldom completely effective and visually harmful even when they are used in the short term or in an “emergency.” It is necessary to understand that physical restraint carries risks, and it is essential to have some criteria/rules to minimize them. Therefore, it is important to consider the ethical principles, the clinical aspects and the individual assessment of the patient during and after physical restraint.

There are numerous studies conducted on the use of physical restraints worldwide. But only scanty studies have been conducted in Nepal about the knowledge and use of physical restraints, despite the fact that physical restraint is widely practised to manage aggressive patients and other psychiatric conditions.

### 2.1 Aim

The researchers aimed at assessing the in‐depth knowledge and use of physical restraint with the existing policy in various selected wards of Tertiary Care Hospital of eastern Nepal. The research questions were as follows: (a) What is the level of knowledge and practice on physical restraint? (b) What are the associating factors that can have an impact on knowledge and practice of physical restraint?

## 3 DESIGN

### 3.1 Method

#### 3.1.1 Research design

A descriptive cross‐sectional research design was adopted in this study.

#### 3.1.2 Setting

This present study was carried out at a Tertiary Care Hospital, Dharan, which lies in the eastern part of Nepal. This institute is a 750 bedded hospital and one of the largest referral centres in the eastern region of Nepal. The study settings selected for this study were ICU/CCU, medical wards, psychiatric wards and emergency units where physical restraint is being practised regularly.

#### 3.1.3 Participants

The study participants were nurses who were working as staff nurse and above. After considering the eligibility criteria and using total enumerative sampling technique, 117 participants were enrolled in the study. Written consent was then obtained from them. Self‐administered questionnaire including socio‐demographic profile, questions concerning knowledge and practice about physical restraints were distributed.

### 3.2 Instruments

It consisted of three sections: Section A was related to socio‐demographic profile, Section B to knowledge questionnaire and under Section C were questions about practice on physical restraints. An extensive literature search was carried out to get an idea on the structure of the knowledge questionnaire about physical restraint and correspondingly was adopted from the research study on the similar topic by Cunha et al. (2016) and Huang, Chuang, and Chiang (2009). The questionnaire was modified in such a way that it fitted the hospital practice and policy. It consisted of 41 items with few items having multiple responses. It was divided into four domains such as patients’ safety, legal and ethical practices, scientific knowledge and quality of care. It is a self‐administered questionnaire. For every correct response, the score of 1 was awarded and for no or incorrect answers 0 was given. The maximum obtainable score was 102. All the correct answers were added and calculated in the percentile form to get the total knowledge scores.

The other part of the questionnaire, which was a self‐administered questionnaire, was related to the practice of physical restraint. The questionnaire was originally developed in Chinese and was modified by Huang (2003) and validity and reliability were established. Cronbach's alpha for reliability of the questionnaire was 0.77. This modified version was adopted from study of Huang et al. (2009). The tool used in this study was to measure degree of correct use of physical restraints, using a 3‐point Likert scale. It consisted of 20 items. Regarding the interpretation, 0 was scored for “never” responses, “sometimes” 1 was scored and “always” 2 was scored, with a total maximum obtainable score of 40. The mean value was used as a cut‐off to interpret as adequate and inadequate practice of physical restraint.

After finalizing the structure of the tools, intensive group discussions were conducted with the selected charge nurses of the hospital to generate further ideas on the knowledge and practice of physical restraint. Finally, the questionnaire was sent to the two psychiatrists and a psychiatric nurse for content validity. Based on the suggestions provided by the experts, the questionnaire was given a final shape.

### 3.3 Ethical approval

This proposal obtained ethical clearance from the Institutional Review Committee (IRC), Code No: IRC/0961/017.

### 3.4 Data analysis

The collected data were checked, reviewed and organized for accuracy and completeness. Editing and coding of data were carried out. All the data were entered in Microsoft Excel 2007 and then transferred to SPSS version 21.0. The data were analysed by using descriptive and inferential statistical methods: taking 95% confidence, 5% permissible error and “*p*” value = 0.05. Descriptive statistics (frequency, percentages, median, mean, standard deviation) were calculated. Inferential statistics such as Pearson chi‐square test and Fisher's exact test were applied to find out the association between socio‐demographic variables and knowledge and practice. Correlation coefficient test was applied to find out the relationship between knowledge and practice regarding physical restraint.

## 4 RESULTS

### 4.1 Description of socio‐demographic profile of the nurses

From the total sample (*N* = 117), most participants (55.6%) were from the age group 18–25 years (mean = 25.84; *SD* 4.94). Regarding educational status, most of the participants (82.1%) have completed their PCL Nursing (certificate course). In case of working area, 33.3% of the nurses were working in ICU/CCU; 30.8% of the nurses were working in medical wards. Nurses working in emergency area were 28.2%, and only 7.7% of the nurses were working in psychiatric ward. Regarding the experience, most nurses (88.9%) were in between 1–8 years (median [IQR] = 2.5 [1.5–2.5]). Most of the nurses (74.4%) had previous exposure to physical restraint (Details are shown in Table 1).

**Table 1:** Socio‐demographic profile of the nurses: *N* = 117

S. No	Content	Frequency	Percentages
**1**	Age in years		
	18–25	**65**	55.6
	≥25	**52**	44.4
	Mean ± *SD *= 25.84 ± 4.94		
2	Gender		
	Female	116	99.1
	Male	1	0.9
3	Educational qualification		
	PCL Nursing (Diploma)	96	82.1
	BN/B.Sc. Nursing (Bachelor)	21	17.9
4	Work area		
	ICU/CCU	39	33.3
	Psychiatric ward	9	7.7
	Medicine	36	30.8
	Emergency	33	28.2
**5**	Year of experience		
	1–8 years	104	88.9
	8–16 years	7	6.0
	16 and above	6	5.1
	Median (IQR) = 2.5 (1.5–2.5)		
**6**	Previous exposure to physical restraint		
	Yes	87	74.4
	No	30	25.6
**7**	Have you received training related to physical restraint		
	No	95	81.2
	Yes	22	18.8

### 4.2 Knowledge questionnaire on physical restraint

The knowledge of physical restraints showed that 47.9% of the nurses scored below the median (median [IQR] = 43 [54–30]) and 52.1% were above the median range. While analysing on the descriptive part of the questions, it was found that most of the nurses (86.3%) correctly answered the question: “Physical restraint is a protective practice of patient's security.” The nurses gave fair responses to the questions like “Physical restraint is a procedure to protect the nurses from any unexpected harms”; 51.3% responded correctly to the questions; 70.9% agreed that “the practice of physical restraint is documented.” Another interesting result is that only 8.5% of the participants agreed with the statement, “physical restraint is a practice that should be banned.” Physical restraint can be applied only in adults; 72.6% nurses responded incorrectly to the question. A detailed description of frequency and percentages of knowledge questionnaire is shown in Table 2.

**Table 2:** Knowledge questionnaire on physical restraint

S. No	Contents	Correct response		Incorrect response		Did not answer	
		*F*	%	*F*	%	*F*	%
	Regarding patient's safety						
1	Physical restraint involves increased risks to the patient	52	44.4	57	48.7	8	6.8
2	Cases of death after physical restraint are common	0	0.0	113	96.6	4	3.4
3	Physical restraint is a protective practice of patient's security	101	86.3	11	9.4	5	4.3
4	Physical restraint is applied in patients at risk of falling	88	75.2	25	21.4	4	3.4
5	Physical restraint is a risk factor for the development of pressure ulcers	91	77.8	21	17.8	5	4.3
6	Physical restraint is applied in patients with						
	Psychotic disorders	69	59.0	11	9.4	37	31.6
	Substances related disorders	56	47.9	17	14.5	44	37.6
	Personality disorders	33	28.2	41	35.0	43	36.8
	Mood disorders	43	36.8	30	25.6	44	37.6
	Restlessness	43	36.8	34	29.1	40	34.2
7	During the application of physical restraint, the patient feels:						
	Revolted	32	27.4	43	36.8	42	35.9
	Frustrated	54	46.2	22	18.8	41	35.0
	Humiliated	52	44.4	23	19.7	42	35.9
	Worried	48	41.0	27	23.1	42	35.9
	Safe	16	13.7	60	51.3	41	35.0
	Regarding legal and ethical practices						
8	Physical restraint is a procedure to protect the nurses from any unexpected harm	60	51.3	55	47.0	2	1.7
9	Physical restraint is a procedure to use in whenever necessary	77	65.8	37	31.6	3	2.6
10	Physical restraint is an act of abuse of authority	16	13.7	89	76.1	12	10.3
11	Physical restraint promotes human dignity	10	8.5	76	65.0	31	26.5
12	Physical restraint of patients is a punitive act of the patient who is uncooperative	**59**	**50.4**	**48**	**41.0**	**10**	**8.5**
13	Physical restraint is a practice that should be banned	10	8.5	80	68.4	27	23.1
14	Physical restraint of patients is an autonomous act of nursing	21	17.9	77	65.8	19	16.2
15	The use of physical restraint by the nurse depends on Physician's order	50	42.7	58	49.6	9	16.2
16	Orders of physical restraint must be reissued by a physician every 4 hr for adults age 18 years and above and every hours for children	68	58.1	19	16.2	30	25.6
17	The practice of physical restraint is documented	83	70.9	18	15.4	16	13.7
18	Physical restraint can be performed						
	By the Nurse	71	60.7	3	2.6	43	36.8
	By the Doctor	68	58.1	8	6.8	43	36.8
	By any Health Professional	44	37.6	29	24.8	44	37.6
19	Physical restraint is a procedure of						
	Medical and nursing	48	41.0	20	17.1	49	41.9
	Nursing	29	24.8	38	32.5	50	42.7
	Psychiatric	53	45.3	15	12.8	49	41.9
20	In Nepal the physical restraint is a procedure						
	Legal	44	37.6	16	13.7	57	48.7
	Illegal	11	9.4	49	41.9	57	48.7
	Regarding scientific knowledge						
21	Physical restraint may be applied at any age	**63**	**53.8**	**47**	**40.2**	**7**	**6.0**
22	Physical restraint can be applied only in adults	22	18.8	85	72.6	10	8.5
23	Physical restraint is applied due to						
	Motor restlessness after failure of other measures	40	34.2	42	35.9	35	29.9
	Risk of falling after failure of other protective measures	69	59.0	13	11.1	35	29.9
	Aggressiveness	70	59.8	12	10.3	35	29.9
	Chemical restraint failure	43	36.8	37	31.6	37	31.6
24	Physical restraint is against the principle of patient autonomy	50	42.7	39	33.3	28	23.9
25	Physical restraint is an old procedure that nurses learned to apply in hospitals	62	53.0	35	29.9	20	17.1
26	Physical restraint is used in case of compulsory hospitalization	15	12.8	90	76.9	12	10.3
27	Physical restraint means						
	Immobilize the patient	39	33.3	35	29.9	43	36.8
	Immobilize and tie the patient	46	39.3	27	23.1	44	37.6
	Tie the patient	27	23.1	44	37.6	46	39.3
28	Support measures used before the restraint are						
	Intravenous medication	67	57.3	20	17.1	30	25.6
	Intravenous fluids	48	41.0	40	34.2	29	24.8
	Feeding by nasogastric intubation	34	29.1	53	45.3	30	25.6
	Catheterization	47	40.2	39	33.3	31	26.5
	Feeding need	38	32.5	48	41.0	31	26.5
	Elimination need	48	41.0	39	33.3	30	25.6
29	The patient's restraint applies						
	Grids	28	23.9	56	47.9	33	28.2
	Physical restraint	78	66.7	12	10.3	27	23.1
	Bed immobilization	54	46.2	35	29.9	28	23.9
	Chemical restraint	59	50.4	30	25.6	28	23.9
	Isolations	23	19.7	65	55.6	29	24.8
30	When applied, physical restraint immobilizes the following body parts						
	Wrists	87	74.4	14	12.0	16	13.7
	Chest	58	49.6	43	36.8	16	13.7
	Arms	59	50.4	42	35.9	16	13.7
	Hands	72	61.5	29	24.8	16	13.7
	Ankles	48	41.0	53	45.3	16	13.7
	Legs	78	66.7	23	19.7	16	13.7
	Pelvis	15	12.8	86	73.5	16	13.7
	Feet	43	36.8	58	49.6	16	13.7
	Knees	36	30.8	65	55.6	16	13.7
	Elbow	32	27.4	69	59.0	16	13.7
	Thighs	16	13.7	85	72.6	16	13.7
31	Physical restraint uses the following materials						
	Bandages	94	80.3	3	2.6	20	17.1
	Sheets	56	47.9	41	35.0	20	17.1
	Cotton/Pads	82	70.1	15	12.8	20	17.1
	Adhesives	53	45.3	42	35.9	22	18.8
	Restraint vests	43	36.8	52	44.4	22	18.8
	Splints	44	37.6	51	43.6	22	18.8
	Stripes	20	17.1	75	64.1	22	18.8
	Regarding quality of care						
32	The practice of physical restraint is an indicator of nursing care quality	57	48.7	51	43.6	9	7.7
33	Physical restraint should be the last resource in the patient approach, focusing on the verbal and chemical restraint first	90	76.9	17	14.5	10	8.5
34	The restraint is applied in patients with:						
	Intravenous devices	35	29.9	41	35.0	41	35.0
	Chest tubes	17	14.5	59	50.4	41	35.0
	Indwelling catheters	21	17.9	55	47.0	41	35.0
35	Physical restraint of patients is a way of managing human resources	40	34.2	60	51.3	17	14.5
36	Physical restraint is the best way to immobilize the patient	51	43.6	58	49.6	8	6.8
37	Physical restraint of patients reduces the hours of nursing care at every turn	37	31.6	64	54.7	16	13.7
38	The number of patients falling in the ward where you work decreased after the practice of physical restraint	76	65.0	25	21.4	16	13.7
39	Physical restraint is an example of good nursing practices	20	17.1	80	68.4	17	14.5
40	Physical restraint is applicable on all wards with:						
	Restlessness	61	52.1	37	31.6	19	16.2
	Fear	19	16.2	79	67.5	19	16.2
	Trauma	28	23.9	70	59.8	19	16.2
	Consciousness changes	49	41.9	49	41.9	19	16.2
	Aggression	81	69.2	17	14.5	19	16.2
	Dehydration	7	6.0	91	77.8	19	16.2
	Cardio vascular accident	8	2.6	90	76.9	19	16.2
	Respiratory depression	3	2.6	95	81.2	19	16.2
	Extrapyramidal symptoms	10	8.5	88	75.2	19	16.2
	Seizures	55	47.0	42	35.9	20	17.1
41	After the physical restraint the following parameters should be evaluated and recorded periodically:						
	Cutaneous integrity	56	47.9	35	29.9	26	22.2
	Symptoms of circulation (arms and legs)	85	72.6	8	6.8	24	20.5
	Colour	79	67.5	14	12.0	24	20.5
	Vital signs	76	65.0	17	14.5	24	20.5
	State of consciousness	75	64.1	17	14.5	25	21.4
	Communication	59	50.4	33	28.2	25	21.4

Abbreviation: *F*, frequency; %, percentage.

### 4.3 Practice questionnaire on physical restraint

The total practice score showed 54.7% as adequate practice (mean = 80.1, *SD* = 7.79) while 45.3% of the nurses considered the practice as inadequate practice. Overall, 68.4% nurses responded “Always” to the question: “when I believe the patient does not require restraint, I will suggest physician to cancel the order.” Likewise to the question: “When I change the restrained patient's clothing, I will check the patient's skin for any signs of irritation” most of the nurses (93.2%) responded “Always.” Likewise, “When understaffed, more patients will be placed on restraint” most of the nurses 76.9% responded “never.” Unexpected outcome from one of the practice questions: “I will document about the physical restraint in all three phases (before, during and after)” about 17.1% nurses responded with “never.” Description of frequency and percentages of practice questionnaire is shown in Table 3.

**Table 3:** The practice of physical restraint use scale *N* = 117

S. No	Contents	Never		Sometimes		Always	
		*F*	%	*F*	%	*F*	%
1	Before resorting to physical restraint, I attempt all other procedures	7	6.0	44	37.6	66	56.4
2	I believe I am justified in physically restraining a patient	14	12.0	64	54.7	39	33.3
3	When I believe the patient does not require restraint, I will suggest the physician cancel the order	8	6.8	29	24.8	80	68.4
4	When restrained patient asks for assistance, I will respond as soon as possible	6	5.1	22	18.8	89	76.1
5	To ensure safety, I will check the restrained patient once every 2 hr	2	1.7	19	16.2	96	82.1
6	When I change the restrained patient's clothing, I will check the patient's skin for any signs of irritation	1	0.9	7	6.0	109	93.2
7	I will explain the reasons for restraint to the patient	3	2.6	15	12.8	99	84.6
8	I will explain the reasons for restraint to the patient's family	3	2.6	4	3.4	110	94.0
9	I will take care of respiration, nutrition, hydration and elimination need	4	3.4	4	3.4	109	93.2
10	I will communicate with the patient while on physical restraint	3	2.6	37	31.6	77	65.8
11	I will maintain patient comfort taking care of normal sleeping positions	5	4.3	12	10.3	100	85.5
12	I will inform the patient before removing restraints	8	6.8	18	15.4	91	77.8
13	I will inform the patient's family before removing restraints	10	8.5	26	22.2	81	69.2
14	Physical restraint is my first choice to control confused or irritated patients	61	52.1	52	44.4	4	3.4
15	I do read hospital policy and related information regarding use of physical restraint	22	18.8	42	35.9	53	45.3
16	When understaffed, more patients will be placed on restraint	90	76.9	20	17.1	7	6.0
17	The facility and staff will attempt measures other than physical restraint to control behavioural problem	25	21.4	57	48.7	35	29.9
18	I will use anything I deem appropriate to restrain the patient	64	54.7	42	35.9	11	9.4
19	I prefer chemical restraint to physical restraint	31	26.5	72	61.5	14	12.0
20	I will document about the physical restraint in all three phases (before, during and after)	20	17.1	32	27.4	65	55.6

Abbreviation: *F*, frequency, %, percentage.

### 4.4 Association of the knowledge and practice score with selected demographic variables

In association tests, significant association was found between the knowledge score and work area in which nurses working in medical wards were found to have significantly high knowledge as compared to nurses working in other areas such as psychiatric ward, ICU and emergency unit. In years of experience, significant association was found higher among nurses who had the experience of 1–8 years. Similarly, with regard to the practice score and selected demographic variables, significant association was found only with working area. In this case as well, significantly high level of practice was found among nurses working in medical wards.

### 4.5 Correlation between knowledge and practice score

The Pearson correlation coefficient test between knowledge and practice of physical restraints score indicated a positive correlation (Table 4). This means that knowledge and practice are interrelated as when knowledge increase a practice of physical restraint will also be improved.

**Table 4:** Correlation between knowledge and practice score

Variables	*N*	Mean & *SD*	*p* value	*r*
Knowledge	117	70.5 ± 7.78	0.002*	0.28
Practice	117	80.1 ± 7.79		

* Significant correlation.

## 5 DISCUSSION

The use of physical restraints is highly debated among health professionals involved caring and managing the patients in the critical situation and in emergencies. Long‐term use of physical restraints can lead to multiple medical, psychological and functional problems. Thus, the nurses need to be updated and they should anticipate risky problems like death, abrasion at restraint site, incontinence of urine and stool, faecal impaction, dehydration due to lack of access to fluid and decreased functional status.

The present study showed that most of nurses were from ICU/CCU and medical wards and few were from emergency and psychiatric wards. This indicated a fair representation from those area who practised physical restraint rather than focusing on only single area. This finding contradicts a study performed by Sujata and Jasbir (2005) in India, where most (61.66%) of the nurses were working in ICU's and only 38.3% of them were working in neurosurgery and emergency wards. This difference in the results may have occurred because many of the researchers were focused on only in critical area rather than on medical wards. In fact, there is evidence of practice of physical restraints in medical wards and psychiatric wards as well.

The first objective of the study was to assess the level of knowledge of physical restraints. It showed that around 53% of the nurses’ knowledge score was above the median range indicating more than 50% of the knowledge questions. Literally, it can be understood that the nurses’ knowledge was good. This similar finding was found in the study done by Cunha et al. (2016).

Regarding the knowledge questions on patients’ safety, several studies reported cases of death in other parts of the world. However, in Nepal the cases of death after physical restraint remain unreported or absent. In this study too, approximately 97% nurses responded that cases of death after physical restraint is uncommon. This finding is supported by the Sonya and Negm (2013) study. However, this study can be identified as one of the studies in Nepal that reports no injury or death after the use of physical restraint. Likewise, regarding indication for physical restraint most nurses, about 60%, responded on psychotic patients and about 40% in restlessness cases. These findings are contradicted with the study of Cunha et al. (2016) where psychotic disorders came up with 91%, substance‐related disorders with 68.6% and mood disorders with 55.1%. This difference in the findings may be because of the difference in educational background. In this current study, most of the sample's educational qualification was Diploma in Nursing.

Similarly, in discussing on the explanatory section of practice questionnaire, more than half of the nurses agreed that documentation is a must in all three phases (before, during and after). This finding is in contradiction to the findings of Mahmoud (2016) whose study showed that less than half of the respondent nurses indicated that they do not record data about physical restraint use in a patient's chart, for example type of restraint used; indication for use; time of application; and the related nursing care. This may be because nurses in tertiary care hospitals are more conscious about the legal and ethical issues which may arise otherwise.

Also, most nurses selected in this present study had already been exposed to physical restraint in their respective areas and because of this reason, during the practice of physical restraint, most nurses were aware and always assessed patients’ skin when they change the patient's clothing. They realized that communication is important while the patient is in restraint. These two findings contradict the findings of Mahmoud (2016), where 46.9% of the nurses did not monitor patients’ skin frequently even in restrained extremities and do not talk with the client during the procedure (38.5%). This difference might have occurred due to the nurses’ place of work and experience. Likewise, regarding the statement: “When under staffed, more patients will be placed on restraint,” approximately 80% of them disagreed with the statement. This supports the result of Mahmoud (2016): around 60% of participants disagreed with the statement that: “the main reason for restraints used in the hospital is shortage of staff.”

Last, a significant positive correlation was found between knowledge and practice scores. A similar finding was reported by Sonya and Negm (2013). There was a significant positive correlation between respondent nurses' practice score and that of knowledge and attitude scores. Similarly, Eskandari et al. (2017) showed a positive correlation between knowledge, attitude and practice of nurses towards physical restraint administered on patients.

### 5.1 Limitations

In this study, data were collected from only one hospital of Eastern Nepal which limits the external validity of the result. Moreover, practice of nurses regarding the use of physical restraint was assessed by a self‐administered questionnaire, which might not reflect the actual behaviour of nurses. The site of practising physical restraints, nurses who worked in psychiatric wards, must have practised the restraint procedure as per the policy and order of the psychiatrist. Nurses who worked in psychiatric wards must document and complete the restraint order form which states the reason to use physical restraint, any alternatives used and frequency of assessment of restrained patients in every work shift while this procedure was not mandatory in other medical wards nurses. The participants’ educational background can make a difference as the questionnaire was structured in a way that it covers all the theoretical aspect where the diploma nurses might not have idea to the level of graduate nurses.

## 6 CONCLUSION

As shown above, it can be concluded that knowledge and practice of physical restraint are interrelated. However, it was surprising to find out that nurses’ knowledge score was just about average when physical restraints were practised regularly by the nurses. More favourable outcomes can be expected if the knowledge gap is narrowed. Moreover, staff in higher positions or with more years of experience could act as role models for the junior staff members so that appropriate guidance can be provided in both the theoretical and practical application of physical restraints. Hospital administrators should plan in‐service education for all nurses working in various wards of tertiary care hospitals. If physical restraint is to be practised, staff nurses must understand not only how to use it properly but also its negative consequences.

## CONFLICT OF INTEREST

There is no conflict of interest in this study.

## AUTHOR CONTRIBUTIONS

This study was conducted in collaboration of four members. NP being the primary author the whole concept was of her and she started with the proposal writing and faced with the ethical review committee for the defence of the proposal. She also did with the data collection process and till the writing of report. SL contributed in editing the whole study and also refining the tool for the study. Also took part in the initial step of data collection like seeking permission with the hospital authority and meeting with the ward In‐charges. GM contributed in the data entry and analysis of the coded data and writing of the results. ES contribution was in the data collection, data coding and data analysing.

## PATIENT CONSENT

No patient was enrolled in this study since this study was focused on the nurses’ knowledge and practice. Hence, patient consent was not required for this study.
